# A role for whey acidic protein four-disulfide-core 12 (WFDC12) in the pathogenesis and development of psoriasis disease

**DOI:** 10.3389/fimmu.2022.873720

**Published:** 2022-09-06

**Authors:** Fulei Zhao, Chen Zhang, Guolin Li, Huaping Zheng, Linna Gu, Hong Zhou, Yuanyuan Xiao, Zhen Wang, Jiadong Yu, Yawen Hu, Fanlian Zeng, Xiaoyan Wang, Qixiang Zhao, Jing Hu, Chengcheng Yue, Pei Zhou, Nongyu Huang, Yan Hao, Wenling Wu, Kaijun Cui, Wei Li, Jiong Li

**Affiliations:** ^1^ State Key Laboratory of Biotherapy and Cancer Center, West China Hospital, West China Medical School, Sichuan University and Collaborative Innovation Center for Biotherapy, Chengdu, China; ^2^ Department of Obstetrics and Gynecology, West China Second Hospital of Sichuan University, Chengdu, China; ^3^ Key Laboratory of Birth Defects and Related Diseases of Women and Children, Sichuan University, Ministry of Education, Chengdu, China; ^4^ Department of Cardiology, West China Hospital, Sichuan University, Chengdu, China; ^5^ Department of Dermatology, Rare Diseases Center, West China Hospital, Sichuan University, Chengdu, China

**Keywords:** psoriasis, keratinocytes, WFDC12, inflammation, retinoic acid

## Abstract

Whey acidic protein four-disulfide core domain protein 12 (WFDC12) has been implicated in the pathogenesis of psoriasis but the specific molecular mechanism is not clearly defined. In this study, we found the expression of WFDC12 protein closely correlated with psoriasis. WFDC12 in keratinocyte might increase infiltration of Langerhans cells (LCs) and monocyte-derived dendritic cells (moDDCs), up-regulating the co-stimulation molecular CD40/CD86. Th1 cells in lymph nodes were higher in K14-WFDC12 transgenic psoiasis-like mice. Meanwhile, the mRNA of IL-12 and IFN-γ in the lesion skin was significantly increased in transgenic mice. Moreover, we found that the expression of the proteins that participated in the retinoic acid–related pathway and immune signaling pathway was more changed in the lesion skin of K14-WFDC12 transgenic psoriasis-like mice. Collectively, the results implied that WFDC12 might affect the activation of the retinoic acid signaling pathway and regulate the infiltration of DC cells in the skin lesions and lymph nodes, thereby inducing Th1 cells differentiation and increasing the secretion of IFN-γ to exacerbate psoriasis in mice.

## 1 Introduction

Psoriasis is a skin-specific autoimmune disease, with excessive thickening of the epidermis, chronic inflammation of the dermis, erythema, and skin scales. According to worldwide statistics, about 2–4% population of the world suffers from psoriasis (1, 2), of which 20–30% will develop into psoriatic arthritis, cardiovascular disease, metabolic syndrome, and other diseases such as inflammatory bowel disease seriously, and the physical and mental health of patients was damaged (1, 3, 4). Several psoriasis immune preparation drugs have entered clinical trials, including immunosuppressants such as interleukin-17 (IL-17) and TNF-α antibodies, which have effective targeting effects but with obvious side effects. For example, Efalizumab targeting the integrin CD11a, and Briakinumab monoclonal antibody targeting the P40 subunit of interleukin-23 (IL-23/P40), can cause severe immune infections, malignant tumors, and other complications (5, 6). In addition, psoriasis has a serious impact on patients, so there is an urgent need to explore effective therapeutic targets for psoriasis.

In the emergence and progression of psoriasis, the skin as a unique regional immune environment area is exposed to numerous environmental variables, such as trauma, stress, and infection. In this region, keratinocytes (KCs) are not only the critical situation in inducing the abnormal activation of the immune mechanism of psoriasis but also the important manifestation of the hyperproliferation of this disease. KCs can release the antimicrobial peptide LL37 and activate plasmacytoid dendritic cells (pDCs) and then promote maturation of myeloid dendritic cells (MDCs) to secrete inflammatory cytokine (7, 8), further inducing adaptive immune activation and promoting the differentiation of naive T cells into T helper type 1 cells (Th1 cells) and Th17 cells in lymph nodes (2). IL-12 as the most critical inflammatory cytokines, secreted by Langerhans cells (LCs) and monocyte-derived dendritic cells (moDDCs), was confirmed to mainly induce Th1 cells, which secreting interferon-γ (IFN-γ) (2, 9–11). Th1 and Th17 cells were recruited by inflammatory cytokines and chemokine to gather in the skin lesions of psoriasis patients. These T cells produce inflammatory factors such as IFN-γ, IL-17, which will in turn act on KCs, vascular endothelial cells, and neutrophils, forming a feedback loop with an amplifying effect in lesion skins (12–14). Among them, the excessive proliferation of KCs eventually leads to epidermis thickness and the formation of scales (15). Neutrophils can activate the accumulation of oxidative stress at the inflammatory site (16). Studies have shown that, after IFN-γ, secreted by Th1 cells, was injected into the non-lesional area of the patient, the expression of inducible nitric oxide synthase (iNOS) increased (17). It can be seen that DC cells (such as LCs, moDDC cells), Th1 and Th17 cells, involved in the immune-inflammatory process to promote the aggravation of immune inflammation, were inseparable from where the KCs were located. Therefore, we imagine that targeting KCs, screening new candidate molecules that are specifically and highly expressed in the skin and are closely related to psoriasis, may have been related to these immune cells to influence pathogenesis and development of psoriasis and will have a broad prospect of the treatment exploitation of this disease.

The early study of our laboratory found that the novel WAP four-disulfide core domain 12 (WFDC12) gene of the WFDC family was highly expressed in the KCs through an integrative approach (18). Several genes including WFDC12 in the WFDC family were located on human chromosome 20q13, which was in the hot spots of psoriasis susceptibility genes (19). The structure of the WFDC family protein was characterized by the core disulfide domain FDC containing eight conserved cysteines, which formed four stable disulfide bonds (19). This domain contains 40–50 amino acid residues, most of these family members are small secret molecules. Among the current 18 members of the WFDC family, the most studied elafin (WFDC14) and secretory leukocyte peptidase inhibitor (SLPI) have been reported to be highly expressed in psoriasis lesions (20–22). SLPI affected the pathogenesis of psoriasis by restricting the formation of neutrophil extracellular traps (NETs) (23) or sensitizing extracellular DNA to stimulate pDCs to secrete IFN-α (22). The serum content of elafin had a significant clinical correlation between psoriasis severity and inflammatory markers (C-reactive protein, erythrocyte sedimentation rate) (24).

A widely accepted notion is that the domain of WFDC proteins has serine protease-inhibitory activity (25–27). The dysfunction of proteases and protease inhibitors may cause various skin inflammatory diseases. For example, the integrin-metalloprotease 17 specifically knocked out in KCs will reduce the activity of transglutaminase in the skin of mice after 2 days of birth and lead to the expression of inflammatory cytokines, such as IL-1β and IL-6, which was up-regulated in skin disease patients (28, 29). The inhibitory molecule Cystatin A inhibited endogenous and exogenous proteases from participating in epidermal structure and biochemical defense mechanisms, which can cause ichthyosis and acre peeling skin syndrome (30, 31). The candidate molecule WFDC12 belongs to the WFDC protein family, with protease inhibitory function, also suggested that WFDC12 may be likely to play an important role in the “skin involved” autoimmune disease psoriasis.

WFDC12 was located at the susceptible site of psoriasis, specific high expression in the KCs; the role and mechanism on the pathogenesis of psoriasis had not yet been reported. Therefore, the role of WFDC12 in psoriasis initially explored the correlation between the severity of psoriasis and the expression of WFDC12. We found that, in the Imiquimod (IMQ)-induced psoriasis-like skin lesion model, K14-WFDC12 transgenic mice, capable of stably expressing WFDC12 in KCs, showed more severe epidermal hyperplasia and inflammatory cells infiltration. We found more increase in infiltration of LCs and moDDCs in lymph nodes with high expression of WFDC12 in KCs of mice. Transgenic mice also had considerably higher levels of Th1 cell differentiation in lymph nodes. The iTRAQ is a high-throughput quantitation approach that enables the simultaneous identification and relative quantification of proteins in up to eight different biological samples of a single experiment with more sensitivity and better accuracy. Based on the iTRAQ proteomic profiling methods, we have initially explored the relevant mechanisms of WFDC12 regulating psoriasis; the results showed that the exacerbated inflammation in K14-WFDC12 mice may be caused by the change of retinoic acid signaling pathway, providing a new theoretical basis for the exploration of new targets and ideas in clinical psoriasis treatment and diagnosis.

## 2 Result

### 2.1 WFDC12 expression positive correlates with psoriasis clinical features

**Figure 1:**
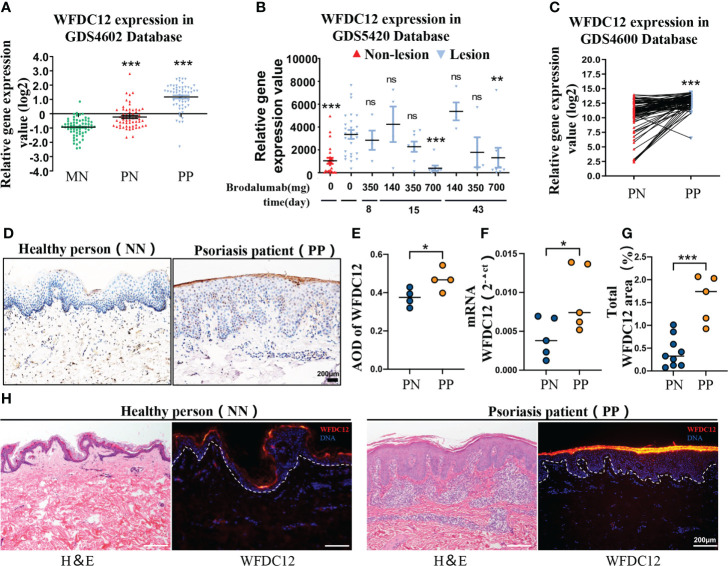
WFDC12 expression positive correlates with psoriasis clinical features. **(A)** WFDC12 expression in skin lesions/non-lesional areas of patients with psoriasis and normal skin based on the GEO database (GDS4602). **(B)** To compare the difference of WFDC12 expression in lesions after IL-17RA antibody treatment based on the GEO database (GDS5420). **(C)** Analyzes the differential expression of paired genes in the lesions and non-lesions of the same psoriasis patient based on the GEO database (GDS4600). **(D)** Immunohistochemical staining of WFDC12 in normal skin tissue of healthy person and skin tissue of psoriasis lesion area. Scale bars, 200μm. **(E)** The expression of WFDC12 in each tissue between each sample according to the average optical density (AOD) (*n* = 4/group). **(F)** RT-qPCR analysis of WFDC12 in normal human skin and psoriasis patients’ skin (*n* = 5/group)**. (G, H)** quantification and IF staining showed WFDC12 presence in healthy human skin (*n* = 8) and skin tissue of psoriasis lesion area (*n* = 5). Scale bars, 200 μm. Dashed lines indicate border between epidermis and dermis. H&E staining of the skin is shown for orientation. Nuclei are stained with DAPI (blue). **(A, B, E–G)** unpaired two-tailed Student’s *t*-test. **(C)** Paired Student’s *t*-test. *M* ± *SD*. *p < 0.05, **p < 0.01, ***p < 0.001. ns, not significance. NN, normal skin tissue of healthy people; PN, skin tissue of non-lesion area of psoriasis patients; PP, skin tissue of psoriasis lesion area.

To identify the differential expression of WFDC12 in psoriasis patients (lesional and non-lesional) and healthy persons, we obtained the publicly available microarray datasets GDS4602, GDS5420, and GDS4600 from the GEO (Gene Expression Omnibus, National Institutes of Health). The expression of WFDC12 was significantly increased in the lesions of psoriasis patients compared with non-lesions and healthy skin tissues (**Figure 1A**). Twenty-five patients with moderate to severe plaque psoriasis were sampled in the lesion areas after the use of different doses of Brodalumab (140, 350, and 700 mg). Brodalumab can improve at least the 70% psoriasis skin lesions of 700 mg treatment groups according to the Psoriasis Area and Severity Index (PASI) for treatment evaluation. It showed a great therapeutic effect on the therapy of psoriasis. At this dosage, as the treatment time increased, the expression of WFDC12 protein presented a downward trend (**Figure 1B**). The expression of the lesion area was higher than that in the non-lesion area for the same patient (**Figure 1C**). We collected clinical skin samples (taken from the West China Hospital) and used immunohistochemical staining to detect the expression of WFDC12 protein in the skin of healthy persons and lesions area of psoriasis patients. We found that WFDC12 expression was higher in psoriatic lesions than in normal skin (**Figures 1D, E**). Moreover, the mRNA level of WFDC12 was higher in the lesions of psoriasis patients relative to healthy skin tissues **(** **Figure 1F** **)**. Immunofluorescence staining of skin sections from both normal and psoriatic patients showed that WFDC12 expression was higher in psoriatic lesions than in normal skin **(** **Figures 1G, H** **).**

### 2.2 IMQ-induced more severer epidermal hyperplasia and inflammatory cells infiltration in K14-WFDC12 transgenic mice than in wild-type mice

**Figure 2:**
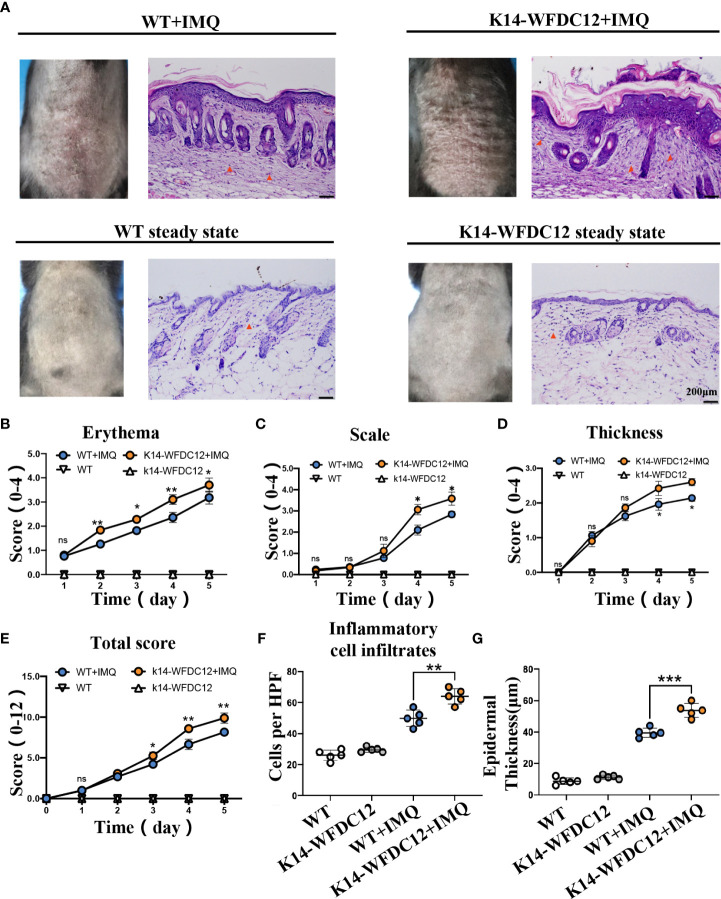
Characterization and evaluation of psoriasis models in K14-WFDC12 transgenic mice and WT mice before and after IMQ-induced. **(A)** Representative photos taken and back skin sections stained with H&E on days 0–5 at steady-state and after IMQ-induced psoriasis model. Scale bars, 50 µm,*n* = 5/group. **(B-D)** PASI score of erythema, scale, and epidermal thickness in the back skin of ​​ mice (0–4 points) at steady state and after IMQ-induced psoriasis model, *n* = 5/group. **(E)** Total PASI score of back skin of mice (0–12 points) at steady state and after IMQ-induced psoriasis model, *n* = 5/group. **(F)** The number of inflammatory cell infiltrates in the dermis at steady state and IMQ-treated. Numbers of dermal inflammatory cells were counted per high-power field from five mice per group. **(G)** epidermal thickness statistics of mice at steady-state and after IMQ-induced psoriasis model, *n* = 5/group. **(B–E)** Unpaired *t*-test was applied to assess the significance of difference between the two groups at the same time points. **(F, G)** One-way ANOVA followed Dunnett was applied to assess the significance of difference among the four group. The data are presented as *M* ± *SD*.*p < 0.05, **p < 0.01, ***p < 0.001. ns, not significance.

To further determine the potential role of WFDC12 in the pathogenesis of psoriasis, first, K14-WFDC12 mice were constructed (**Figures S1A–C**).Compare with wild-type (WT) mice, the WFDC12 expression increased in K14-WFDC12 transgenic mice in back skin **(** **Figure S1D**). There was no significant difference between K14-WFDC12 transgenic mice and WT mice in appearance, body weight, skin structure, and pathological characteristics at a steady state **(** **Figure S1E–I** **)**. In our experiment, we found that, compared with the IMQ-induced WT mice model, the IMQ-induced K14-WFDC12 transgenic mice had a severer psoriasis-like lesion and inflammation (**Figure 2A**). The scores of each group of mice, from 0 to fifth day, were shown in **Figures 2B-E**. The PASI scores showed that the erythema of the K14-WFDC12 transgenic mice changed more significantly on Days 2–5 than that of WT mice (**Figure 2B**), and the scales and epidermal thickening of transgenic mice were more statistically evident on Days 3–5 than those of WT mice (**Figures 2C, D** **)**. The total PASI scores were not significantly different from the transgenic mice group and WT mice group in the initial two days but were statistically significant from Day 3 to 5 (**Figure 2E**).

After IMQ modeling on the fifth day, the epidermal thickness and immune cell infiltration in the H&E staining were statistically analyzed. We found that, compared with WT mice, the inflammatory cells in transgenic mice significantly infiltrated into the skin after IMQ treatment. The number of immune cells per HPF of skin lesion area of K14-WFDC12 transgenic mice models was about 1.5 times that of the WT mice model **(** **Figure 2F** **).** The average value of the epidermal thickness of each group of mice was statistically plotted **(** **Figure 2G** **)**. There is no statistical difference in the thickness of the back skin between K14-WFDC12 transgenic mice (11.25 ± 1.30 µm, *N* = 5) and WT mice at the steady state (9.75 ± 2.17 µm, *N* = 5). After IMQ-induced psoriatic lesion, the epidermal thickness of the K14-WFDC12 transgenic mice models (39.50 ± 1.71 µm, *N* = 5) was significantly increased than that of the WT mice model (53.75 ± 2.53 µm, *N* = 5). These results showed that K14-WFDC12 transgenic mice had a higher degree of psoriasis-like inflammation on the back of the skin than WT mice after IMQ-induced psoriasis lesion.

### 2.3 Much more infiltration of LCs in epidermis and lymph node of K14-WFDC12 transgenic mice after IMQ-induced

**Figure 3:**
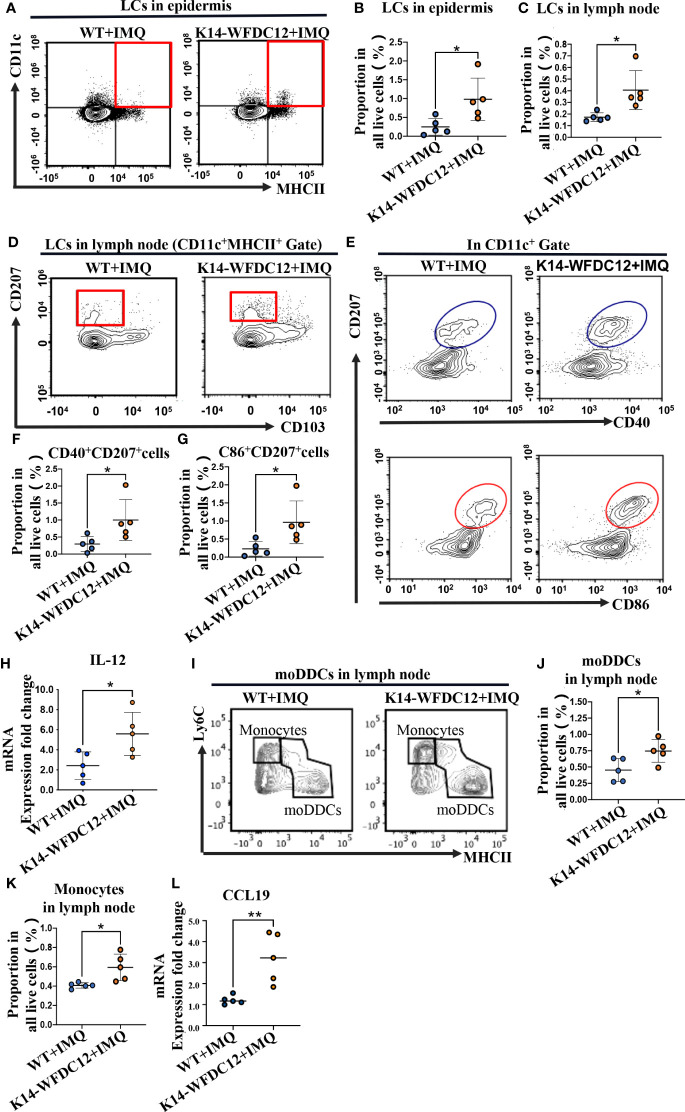
Infiltration of LCs and moDDCs be detected by flow cytometry after IMQ-induced psoriasis-like mice. **(A, B)** Representative and quantification of FACS analysis of LCs in the epidermis after IMQ application, *n* = 5/group. **(C**, **D)** Representative and quantification of FACS analysis of LCs in lymph nodes after IMQ application, *n* = 5/group. **(E–G)** Representative and quantification of FACS analysis of CD40/CD86 expression of LCs in the epidermis after IMQ application, *n* = 5/group. **(H)** IL-12 mRNA expression level of psoriasis-like lesion areas in K14-WFDC12 transgenic mice and WT mice. **(I–K)** Representative and quantification of FACS analysis of moDDCs in lymph nodes after IMQ application, *n* = 5/group. **(L)** CCL19 mRNA expression level of psoriasis-like lesion areas in K14-WFDC12 transgenic mice and WT mice. All the data are shown in this and the figures below came from samples from individual mice unless stated otherwise. The data are presented as *M* ± *SD*. *p < 0.05, **p < 0.01. (unpaired Student's *t*-test).

LCs were located in the outermost layer of the skin and will be the first to respond to infection and physical and chemical damage. They not only could expand their numbers or through monocyte differentiation from peripheral blood and exert immune activation functions *in situ* but also migrate to draining lymph nodes to active differentiation of T cells after mutation (32, 33). It was demonstrated that motivated LCs expressed higher levels of maturation markers, such as CD40/CD80, CD86 (32). For analysis, we prepare single-cell suspensions for flow cytometry detection. The LCs in the epidermis were identified based on the expression of CD45, MHCII, and CD11c. At the steady state, there was no significant statistical difference of LCs in the epidermis between transgenic mice and WT mice **(** **Figures S2A, B** **).** After IMQ-induced psoriasis, the number of LCs of K14-WFDC12 transgenic mice in the epidermis increased higher than that in WT mice **(** **Figures 3A, B** **)**. We also detected the infiltrated LCs in the lymph node. In the inflammatory state, there were a variety of types of dendritic cells in the lymph node. Therefore, the LCs should be characterized further based on the expression of additional markers. LCs were separated from MHCII^+^CD11c^+^ cells based on their expression of CD11b, CD207/Langerin, and CD103. Finally, we identified LCs in lymph nodes as CD11b^+^CD207^+^CD103^-^. There was no difference in the number of LCs between two types of mice without IMQ application **(** **Figures S2C, D** **)**. However, the results showed that LCs of K14-WFDC12 transgenic mice infiltrated much more than those of WT mice by IMQ application **(** **Figures 3C, D** **)**.

At a steady state, there was only a small portion of LCs expressing CD40, CD86 in epidermis and lymph nodes, and no different expression level was detected in K14-WFDC12 transgenic mice and WT mice (**Figures S2E–G**). However, the expression of CD40 and CD86 was numerically increased by IMQ application, and LCs of transgenic mice showed more CD40 and CD86 expressing (**Figures 3E-G**). We also detected higher IL-12 expression levels in lesion areas of K14-WFDC12 mice (**Figure 3H**), which may be secret by LCs in the IMQ-induced psoriasis model.

### 2.4 The infiltration of moDDCs and monocytes was increased in K14-WFDC12 transgenic mice after IMQ-induced

moDDCs are differentiated from Ly6C^+^ monocytes. Under normal physiological conditions, the quantity of these cells is quite low. However, these cells expand and aggregate in large numbers when inflammation occurs. They were identified as the principal contributors to inflammation in psoriasis-like mice (34). We isolated lymph nodes from each group of mice, preparing single-cell suspension for flow cytometric antibody staining. The expression of CD11b and MHCII was identified in CD45^+^ cells. CD64 and Ly6C were recognized as the surface markers of monocyte-derived cells, such as monocytes and macrophages, so we further distinguished the monocyte-derived cells among them (**Figure S2H, Gate B**). Mer tyrosine kinase (MerTK) and CD64 are the most common antigens that distinguish macrophages, so the CD64^+^/MerTK^+^ cells were identified as macrophages. And the CD64^-^/MerTK^-^ cells were further used to identify monocytes and moDDCs by a combination of Ly6C and MHCII. Finally, we identified Ly6C^hi^MHCII^-^ cells as the monocytes, and Ly6C^int/low^MHCII^hi^ cells as the moDDC cells (**Figure S2H**). After the application for IMQ, we detected the infiltration of moDDCs in the lymph node. The results showed that more numbers of moDDCs infiltrated into K14-WFDC12 transgenic mice (**Figures 3I-K**). Meanwhile, expression of chemokines CCL19 in the skin lesion area of K14-WDFDC12 mice increased after IMQ-induced **(** **Figure 3L** **).** We speculated that WFDC12 may affect immune activation, which was closely relative to LCs and moDDCs.

### 2.5 Th1(IFN-γ-secreting) differentiation is up-regulated in lymph nodes of K14-WFDC12 transgenic mice than in WT mice after IMQ-induced

**Figure 4:**
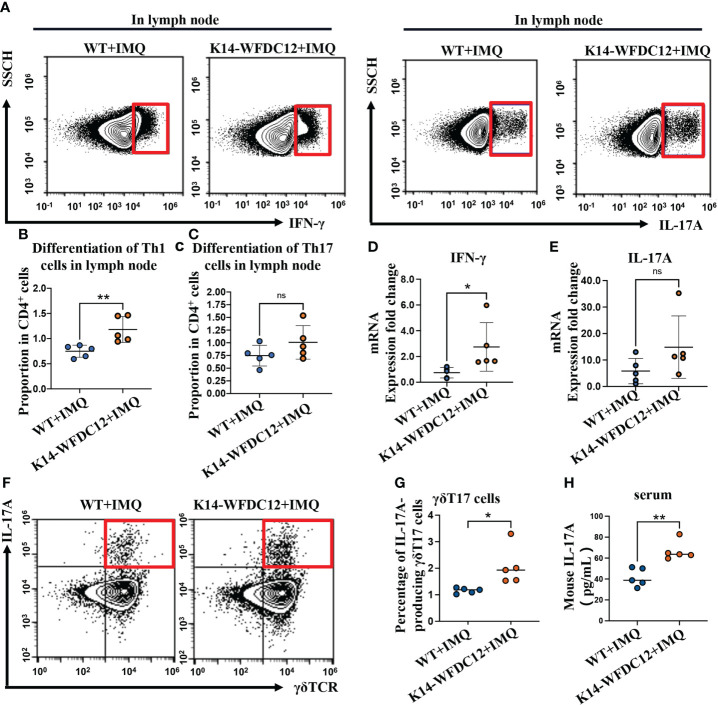
Th1, Th17 and IL-17A-producing γδT17 cells be detected in K14-WFDC12 transgenic mice and WT mice after IMQ-induced psoriasis model. **(A)** Representative of Th1 cells secreting IFN-γ and IL-17-producing cells (Th17 cells) in the lymph nodes of mice after IMQ-induced. **(B**, **C)** Quantification of Th1 cells and Th17 cells in the lymph nodes of mice (proportion of CD4^+^ cells Gate), *n* = 5/group. **(D**, **E)** IFN-γ and IL-17A mRNA expression level of psoriasis-like lesion areas in K14-WFDC12 transgenic mice and WT mice. **(F**, **G)** Representative FCM images and quantification indicated percentages of IL-17A–producing γδT17 cells in lymph nodes of K14-WFDC12 transgenic mice and WT mice treated with IMQ, *n* = 5/group. **(H)** ELISA assessed the levels of IL-17A in serum of K14-WFDC12 mice and WT mice treatment with IMQ, *n* = 5/group. All the data are shown in this and the figures below came from samples from individual mice unless stated otherwise. The data are presented as *M* ± *SD*. **p < 0.01. ns, not significance. (unpaired Student’s *t*-test).

Mature DC cells proliferate and migrate into lymph nodes, polarizing naïve T cells to differentiate into Th1 and Th17 cells, which secret critical pro-inflammatory cytokines IL-17 and IFN-γ in pathogenesis and development of psoriasis (35, 36). We next examine the effect of the WFDC12 on Th1 and Th17 cells differentiation. The murine lymph nodes after IMQ-induced psoriasis were collected for the preparation of single-cell suspension on Day 5; the proportion of Th1 and Th17 cells was analyzed by flow cytometry. We found that there was no difference in the proportion of Th1 cells (CD3^+^CD4^+^IFN-γ^+^) between the K14-WFDC12 transgenic mice and WT mice in the lymph nodes under steady state, and no difference in Th17 cells (CD3^+^CD4^+^IL-17^+^) was detected either (**Figures S3A–D**). After IMQ-induced psoriasis, the proportion of Th1 cells that secrete IFN-γ (CD3^+^CD4^+^IFN-γ^+^) in the lymph nodes of K14-WFDC12 transgenic mice was higher than that of WT mice (**Figures 4A, B** **)**, whereas Th17 cells that secrete IL-17 (CD3^+^CD4^+^IL-17^+^) has no statistically significant differences between two IMQ-induced psoriasis groups (**Figures 4A, C** **)**, but compared with WT mice, the differentiation of Th17 cells in lymph nodes of K14-WFDC12 transgenic mice showed an overall trend of increase. The mRNA expression levels of cytokines IFN-γ and IL-17 in the lesion area of two modeling groups were detected. The inflammatory cytokines IFN-γ was more significantly increased from K14-WFDC12 transgenic mice than in WT mice, and there was no difference in the mRNA expression level of IL-17A (**Figures 4D, E**). Because the high expression of IL-17 in psoriasis may come from γδT cells, we detected IL-17A–producing γδT in lymph nodes of WT and K14-WFDC12 transgenic mice. There was no statistically significant difference in the proportion of IL-17A–producing γδT17 cells in lymph nodes in steady state (**Figures S3E, F**), but there was statistically significant difference in the proportion of IL-17A–producing γδT17 cells in lymph nodes after IMQ-induced psoriasis mice model (**Figures 4F, G** **)**. Compared with WT mice, serum IL-17A level in IMQ-treated K14-WFDC12 mice increased and the difference was statistically significant **(** **Figure 4H** **)**. It was indicated that WFDC12 may induce the differentiation of Th1 cells in the lymph nodes by regulating the infiltration of DC cells in the skin lesions and lymph nodes (including LCs and moDDCs) and increase the secretion of IFN-γ in the skin lesions, thereby exacerbating the pathogenesis of psoriasis.

### 2.6 The retinoic acid–related pathway was changed from K14-WFDC12 psoriasis-like mice comparing with WT mice

**Figure 5:**
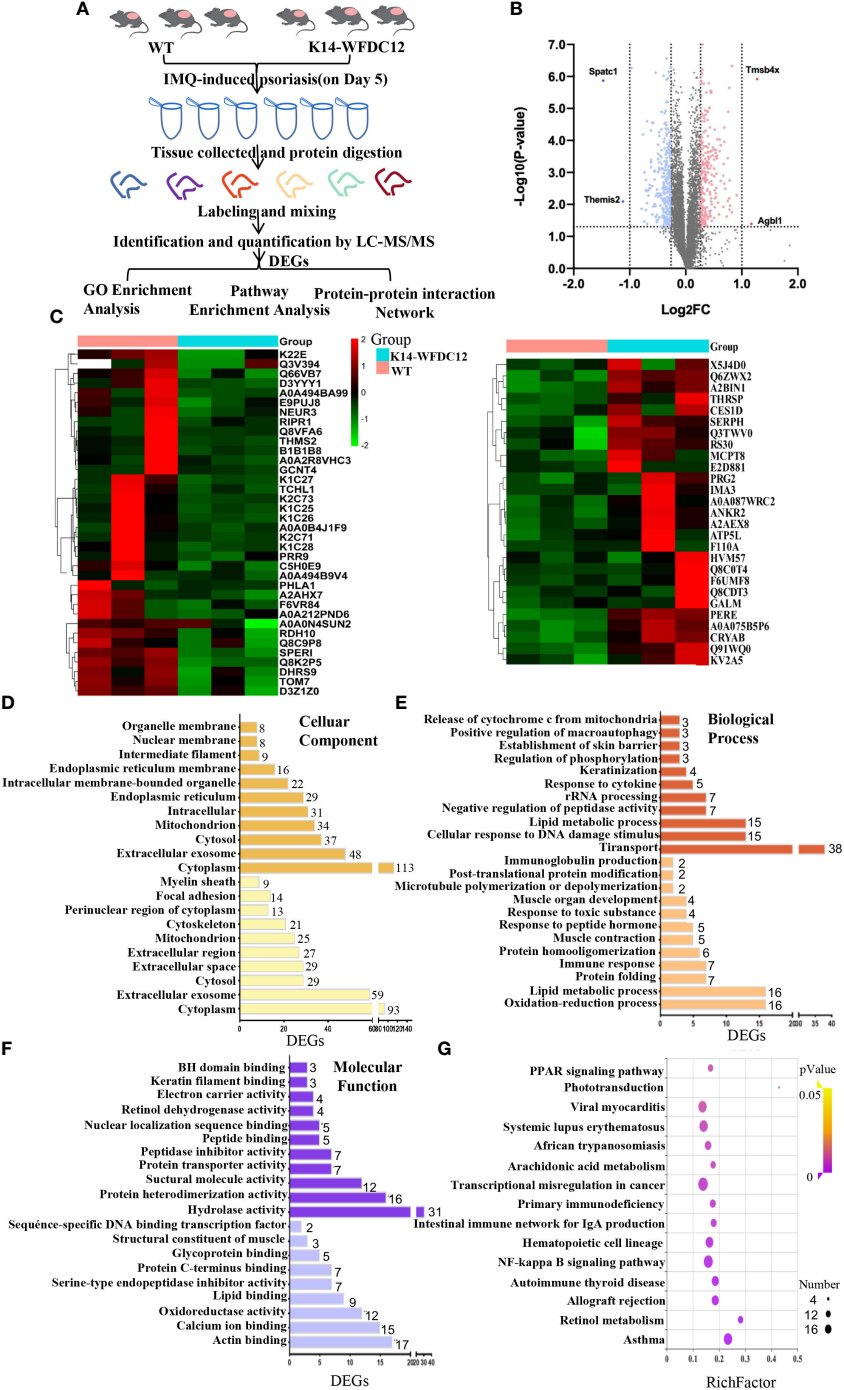
iTRAQ-based proteomics profiling in K14-WFDC12 transgenic mice and WT mice after IMQ-induced psoriasis. **(A)** iTRAQ-based proteomic work-flow in the back skin lesions of mice after IMQ-induced psoriasis model. **(B)** significantly DEGs volcano map (K14-WFDC12 transgenic group vs WT group). The *X*-axis of the figure is the multiple of protein difference (take log_2_), and the *Y*-axis is the corresponding -log_10_ (*P*-value). **(C)**Heat map images show differential genes in skin lesions of WT and K14-WFDC12 mice after IMQ application. Blue indicates low expression level and red indicates high expression level.In all, 520 genes were significantly altered with *P* < 0.05. **(D–F)** GO analysis results of cellular components, molecular functions, and biological processes of DEGs. **(G)** Bubble diagram of the KEGG pathway significantly enriched (K14-WFDC12 transgenic group vs. WT group) after IMQ application. The *X*-axis enrichment factor (RichFactor) presents the number of differential proteins annotated to the pathway divided by all the identified proteins annotated to the pathway. The larger value means that the larger the proportion of differential proteins in the pathway is. The size of the dot in the figure represents the number of differential proteins annotated to the signaling pathway.

WFDC12 may affect the certain signaling pathway to protease inhibition and lead to aggravation of inflammation in the psoriasis immune microenvironment, which has not been reported yet. We explored the proteome profiles by iTRAQ-based quantitative proteomics workflow in the back skin lesions of K14-WFDC12 transgenic mice and WT mice after the IMQ-induced psoriasis model (**Figure 5A**).

In our experiment, the cleavage peptide solution to WT groups and K14-WFDC12 transgenic group (three samples in each group) were labeled with isotope labels (**Table 1**). Under the filter standard “1% FDR,” a total of 45,289 peptides and 7,342 proteins were identified (**Table 2**). The lengths of most identified peptide lengths were 8–12 amino acids, the 914 identified proteins were characterized by more than 11 unique peptides, and the protein mass of identified proteins mainly varied from 20–50 KDa, especially 100 KDa **(** **Figure S4** **)**. The differential expression genes (DEGs) were detected, and the relative rates of change were analyzed. In the case of biological repetitions unmatched, the condition for screening for differential proteins is folding change more than 1.2 or less than 0.83 (the average of the ratios of all comparison groups) and *p* < 0.05 (*t*-test of all comparison groups). Among them, compared with the WT group, there were a total of 296 differential genes significantly down-regulated and a total of 224 differential genes significantly up-regulated in the K14-WFDC12 transgenic group **(** **Figures 5B, C** **)**.

**Table 1:** i-TRAQ labeling samples.

DATA1_Sample	DATA1_Label
WT1	113
WT2	114
WT3	115
K14-WFDC12-1	116
K14-WFDC12-2	117
K14-WFDC12-3	118

**Table 2:** The result of i-TRAQ-based proteome identification.

Sample name	Total spectra	Spectra	Unique Spetra	Peptide	Unique Peptide	Protein
Mus_musculus	572526	101203	86632	45289	41873	7342

#### 2.6.1 Gene ontology analysis

To understand the specific functions of these differential proteins, we first performed Gene ontology (GO) analysis on these proteins encoded by 224 up-regulated differential genes and 296 down-regulated differential genes. The GO terms with* p* < 0.05 were recognized as significantly enriched by differential genes encoded proteins. Through cellular components analysis of up-regulated and down-regulated differential proteins, the results showed that most of the components of these proteins were located in the cytoplasm, extracellular, exosomes, cytosol, and so forth (**Figure 5D**). Analyzing the biological process of these differential proteins, we found that the up-regulated proteins were closely related to the oxidation-reduction process (GO:0055114), lipid metabolism (GO:0006629), protein folding process (GO:0006457), and immune response (GO:0006955), and the down-regulated proteins mainly focus on transport (GO:0006810), lipid metabolic process (GO:0006629), response to cytokine (GO:0034097), and skin barrier (**Figure 5E**). As for the molecular function, the up-regulated differential proteins mainly participated in lipid binding (GO:0008289), calcium ion binding (GO:0005509), glycoprotein binding (GO:0001948), protein C-terminal binding (GO:0008022), actin binding (GO:0003779), and so forth. The biological process of down-regulated differential proteins was mainly involved in peptide binding (GO:0042277), nuclear localization sequence binding (GO:0008139), keratin filament binding (GO:1990254), retinol dehydrogenase (GO:0004745), and so forth (**Figure 5F**). These results indicated that KCs specific-expressed WFDC12 in transgenic mice may influence lipid metabolism, oxidation-reduction reaction, inflammatory response, and skin barrier related molecules when applying IMQ-induced psoriasis, which resulted in more severe epidermal hyperplasia and inflammation response than WT mice.

#### 2.6.2 String analysis of protein–protein interaction

**Figure 6:**
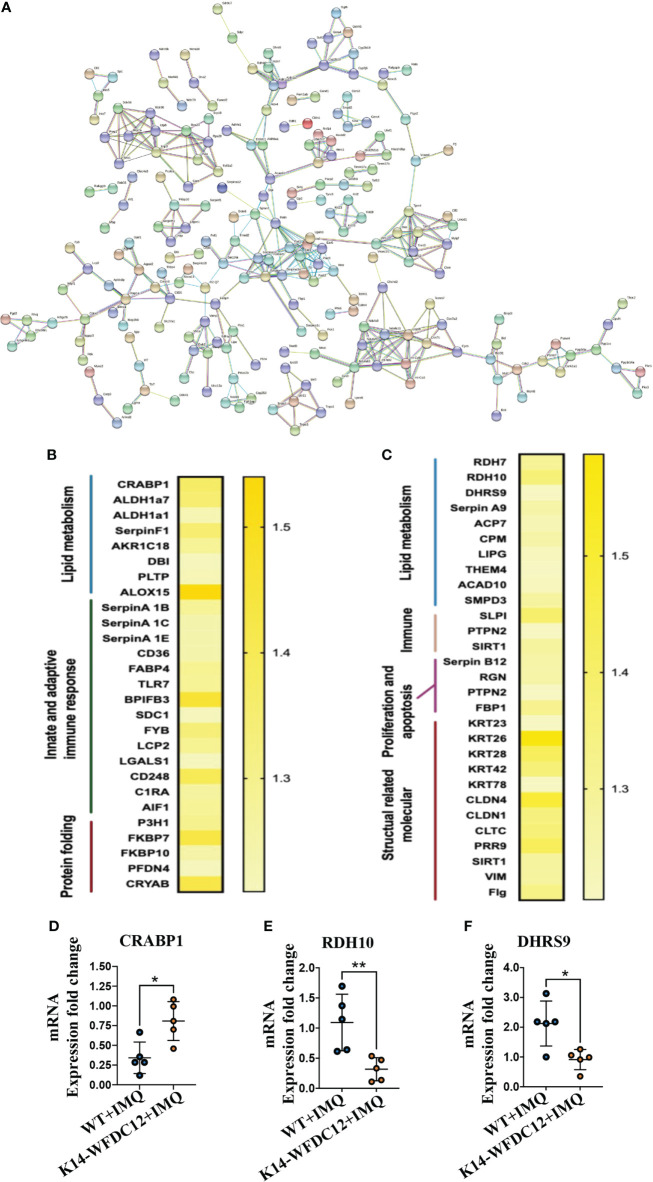
Protein–protein Interaction and heat-map of DEGs after IMQ-induced psoriasis. **(A)** PPI networks of DEGs in psoriasis-like lesion of mice. **(B**, **C)** Heat map of the main differential genes up-regulated and differentially down-regulated. **(D–F)** CRABP1, RDH10 and DHRS9 mRNA expression level. All the data shown in this and the figures below came from samples from individual mice unless stated otherwise. The data are presented as *M* ± SD. *p < 0.05, **p < 0.01. (unpaired Student’s *t*-test).

The protein–protein interaction (PPI) of DEGs between lesion areas of two experimental groups of mice was predicted by the String database (**Figure 6A**). In this network, these proteins encoded by differential genes were mainly included within metabolism (MMU-1430728) and innate immune system (MMU-168249) Reactome Pathways. It includes retinol metabolism, the peroxisome proliferator-activated receptor (PPAR) signaling pathway, and glycan degradation.

From the String results, we found that the Serpin family proteases were extensively included in the differential proteins encoded by the DEGs. The up-regulated genes are SerpinA1B and SerpinA1C, which belong to the SerpinA1 gene encoding different precursor proteins of SerpinA1, and the down-regulated differential genes are SerpinA9 and SerpinB12. Clinical studies have confirmed that the concentration of SerpinA1 in the serum of psoriasis patients with different stages of pathological characteristics has changed (37), and the expression of various isoenzymes of SerpinA1 was also found in these patients (38). SerpinB12 of the same family may take part in protecting epithelial cells and has a pivotal barrier protection function (39). At present, there is no evidence to show the direct action of SerpinA1 family proteins in the occurrence and development of psoriasis, and the relationship between WFDC12 molecules and Serpin family proteins remains to be further proved.

Many differential proteins participated in the T-cell activation related signaling pathway **(** **Figures 6B, C** **)**, including Toll-like receptors 7 (TLR7), FYN-binding protein, and lymphocyte cytoplasmic protein 2 (LCP2), lectin galactosidase binding soluble 1 (Lgals1), and CD248. The allograft inflammatory factors C1RA, which positively regulate the proliferation and migration of T cells and involved in the classical pathway of complement activation, were detected to be up-regulated in transgenic mice. A large number of the down-regulated differential genes encode structural activity-related molecular proteins, which mainly include keratin 26 (KRT26), keratin 28 (KRT28), and keratin (KRT42), claudin 4 (CLDN4), claudin 1 (CLDN1), vimentin (VIM), and filaggrin (Flg). From the above information, we can analyze that there may be activation of lipid metabolism pathways and T-cell–related immune signaling pathways in the pathogenesis of K14-WFDC12 transgenic mice, and cause the downregulation of structural-related protein molecules, which affects cell proliferation and differentiation and other functions.

#### 2.6.3 Kyoto Encyclopedia of Genes and Genomes (KEGG) pathway analysis

To obtain the DEPs information of functional pathways, we further utilize the KEGG database to explore signaling pathways. Here, the top 10 KEGG pathways that we presented (*p* < 0.05) may give some clues of transduction pathways in which the DEGs were possibly taken participate in **Figure 5F**. The signaling pathways with significant differences between the experimental group and control group were as follows: (1) lipid metabolism–related signaling pathways: PPAR signaling pathway (mmu:03320), phototransduction signaling pathway (mmu:04744), arachidonic acid signaling pathway (mmu:00590), retinoic acid signaling pathway (mmu:00830); (2) immunology-related signaling pathway: NF-κb signaling pathway (mmu:04064)); (3) immune-related diseases: systemic lupus erythematosus (mmu:05322), asthma (mmu:05310), autoimmune thyroid diseases, and so forth. Among them, systemic lupus erythematosus and autoimmune thyroid disease are closely related to the immune process.

Combined with the previous analysis, it was worth noting from the KEGG pathway bubble chart that the retinoic acid signaling pathway was with a high Rich Factor **(** **Figure 5G** **)**. After IMQ application, the differentially expressed CRABP1 genes were significantly up-regulated in the WFDC12 experimental group than in the WT mice; the significantly down-regulated differential genes are RDH7, RDH10, and DHRS9 **(** **Figures 6B, C** **)**. They all occupied a crucial role in the signal pathway of retinoic acid synthesis and metabolism. It suggested that specific high expression of WFDC12 in KCs of mice changed the pathways of retinoic acid synthesis and metabolism in the back skin lesions, which may result in regulating the process of activated immune regulation and aggravated severe epidermal hyperplasia.

### 2.7 The expression of inflammatory cytokines and retinoic-acid signaling pathway-related molecules were changed in lesion areas of K14-WFDC12 transgenic mice after IMQ-induced

To further confirm the role of highly expressed WFDC12 in KCs of mice, we examined the mRNA expression levels of cytokines IL-17A and IFN-γ. In lesion areas of back skin, the expression of IFN-γ, IL-12, and CRABP1 was up-regulated **(** **Figures 3H**, **4D** **,** **6D** **)**; the RDH10 and DHRS9 were down-regulated **(** **Figures 6E, F** **)**, whereas the IL-17A mRNA was expressed approximately at the same levels within two groups **(** **Figure 4E** **).**

## 3 Discussion

We previously constructed an *in vitro* M5 stimulation model with great clinical relevance. Through this model, transcriptome sequencing and screening were performed to successfully sort out new candidate molecule WFDC12, which was specifically and highly expressed in KCs and closely related to psoriasis susceptibility sites. The expression of WFDC12 protein in the psoriasis-like lesions area of ​​WT mice was increased *in vivo (* 18). To further explore the clinical relevance of the WFDC12 and psoriasis disease, we analyzed the clinical database and found that the expression of WFDC12 was increased with the aggravation of the pathological characteristics of psoriasis. The AP-1 is the transcription factor-binding sites in the gene promoter that initiates the activation of WFDC12 gene expression, which plays an important function as a disease-related transcriptional regulatory activator in psoriatic KCs (40), and continuously up-regulated small proline-rich protein 1A (SPRR1A0), gap junction protein alpha 1 (GJA1), and early growth response 1 (EGR1) genes expression in damaged skin. It appeared that AP-1 acted as a transcriptional activator of disease-related genes in psoriatic KCs, and genomic evidence suggested that AP-1 regulated genes in additional cells. We speculate that the upregulation of WFDC12 expression may be related to the regulation of gene expression by this transcription factor site. During the pathogenesis of psoriasis, the activation of AP-1 transcriptional regulatory factors may lead to the upregulation of WFDC12 molecule expression.

In the activation of immune cells, protease inhibitors serve a regulatory role. In a type II collagen-induced inflammatory model, LCs treated with protease inhibitors can increase T cells in the incidence and progression of arthritis (41). Promoted by the keratin 14(K14) promoter, WFDC12 is specifically highly expressed in the KCs of transgenic mice. There was increased in infiltration of dendritic cells in the lesions area and lymph node, as did IL-12 release in the area of the lesion after IMQ-induced. Meanwhile, after IMQ-induced, WFDC12 overexpression in KCs increased the expression of CCL19 chemokines in skin lesions and then activated dendritic cells. We speculate that the WFDC12 may regulate the immune system by impacting the infiltration of LCs and moDDC cells in mice after the IMQ-induced psoriasis model. It has been reported that LCs can expand and migrate to lymph nodes to promote inflammation after IMQ modeling 48 h (32), which can release IL-12 to increase the polarization of Th1 in lymph nodes (42). It has been shown that moDDC cells may also drive adaptive immunity to generate a high quantity of IL-12 (43, 44) and that IL-12 can further promote Th1 differentiation and aggravate psoriasis (45). In our experiments, we discovered that Th1 cells of K14-WFDC12 mice infiltrated the lymph nodes substantially more than that of WT mice and that IFN-γ secretion was much higher. Furthermore, after IMQ-induced, IL-17A-producing γδT17 cells were increased in lymph nodes of K14-WFDC12 transgenic mice and serum IL-17A levels were also elevated.

Clinical investigations have demonstrated that psoriasis patients have a significantly increased number of Th1 cells, implying that Th1 plays a key role in the disease progression (46, 47). Under the influence of chemokines released by KCs, polarized Th1 in the lymph nodes can migrate to skin lesions and release the pivotal inflammatory cytokines IFN-γ and others, then aggravating psoriasis (46). It was suggested that WFDC12 may promote the infiltration of LCs in the epidermis and lymph nodes, resulting in increased IL-12 production, hence, enhancing the differentiation of the Th1 cells in the lymph node and the IFN-γ secretion in the lesions area of the IMQ-induced psoriasis model.

To investigate the relevant mechanisms of WFDC12 in psoriasis, the iTRAQ-based proteomics profiling was performed to compare the expression of the different genes in the back skin lesions of WFDC12 transgenic mice and WT mice after IMQ treatment, discovering significant alterations of genes expression that was relative to the retinoic acid signaling pathway in transgenic mice based on the GO analysis, string analysis, and KEGG signaling pathway results, including CRABP1, DHRS9, and RDH10. CRABP1 expression rose; however, RDH10 and DHRS9 expression dramatically dropped. The results of Kalinina P et al. showed that WFDC12 is specifically expressed in terminally differentiated KCs and regulates epidermal serine protease activity (48). Unexpectedly, we performed GO analysis on 520 differential proteins. As for the molecular function, the up-regulated differential proteins participated in serine-type endopeptidase inhibitor activity (GO:0004867) and endopeptidase inhibitor activity (GO:0004866). The biological process of down-regulated differential proteins was involved in serine-type endopeptidase inhibitor activity (GO:0004867) and hydrolase activity (GO:0016787) (**Figure 5E**). The results obtained by our proteomics are consistent with those of Kalinina P et al.

Some binding and transport proteins in the retinoic acid signaling pathway influence retinoid absorption and transport, anabolism, and nuclear transport. Retinol to all-trans retinoic acid (ATRA) production entails two critical steps: redox conversion of retinol to retinal, followed by further conversion to retinoic acid. The conversion of retinol to retinal, which involves the participation of enzymes such as retinol dehydrogenase RDH10 and DHRS9, is the first stage in the creation of ATRA. These enzymes perform an important redox function in the essential phases of ATRA synthesis. In the proteome profiling data of the IMQ-induced psoriasis model, the expression of RDH10 and DHRS9 was down-regulated in the lesion of K14-WFDC12 transgenic mice, meaning that a critical step in the creation of retinoic acid ATRA was blocked, resulting in a decrease in retinoic acid ATRA synthesis.

In addition to the synthesis of retinoic acid, retinoic acid catabolism is regulated by a various range of proteins. FABP gene family transport-binding proteins and P450 enzymes are involved in the catabolic process of ATRA. FABP gene family members that play a crucial role in binding traffickings, such as CRABP1, can bind to ATRA and induce ATRA catabolism to modify transcriptional regulation (49, 50). The combination of CRABP1 and ATRA can cause ATRA to be catabolized by the oxidase in the P450 enzyme system at a lower Km value and a faster pace (51), indicating that CRABP1 can regulate the reduction of ATRA concentration. We discovered an increase in the expression of CRABP1 protein in the back lesion area of K14-WFDC12 transgenic mice, implying that ATRA metabolism was increased and intracellular retinoic acid concentration was decreased in the back lesion area of K14-WFDC12 transgenic mice.

Extensive research has been conducted on the regulation of retinoic acid production and metabolism signaling pathways on immune cells. Previous research revealed that the proportion of LCs in the skin area was significantly reduced after applying the clinical retinoic acid-derived drug Etretinate, which was locally treated in the back skin of mice for four consecutive weeks, and the concentration of the drug was 0.5 and 1.0% (52). The mechanism of the therapeutic retinoic acid-derived medicine Tazarotene (Tazarotene) in psoriasis was discovered to selectively activate RAR subtype molecules (53), and regulate immune cell activation, therefore playing the immunosuppressive role in the development of psoriasis. The low concentration of RA binds to the CRABP family members in the retinol signaling pathway in immune cells and was transported into the nucleus (54, 55), inhibiting the transcriptional regulatory signaling pathway of the transcription factor retinoid acid receptor and retinoid X receptor (RAR-RXR), thereby damaging the maturation of LCs (56). In anti-tumor immunity, retinoic acid has also been shown to suppress the RAR signaling pathway in the immunological milieu, hence, improving the anti-tumor immune response. The results of mice or human monocytes treated with RA showed that RA-induced RAR transcription regulation can down-regulate the expression of IRF4, a gene related to moDC differentiation, and inhibit the differentiation and maturation of monocytes into monocyte-derived DC cells, thereby inhibiting CD4^+^ T cells from secreting secretion of IFN-γ (57). Reduced retinoic acid indeed inhibits the development of LCs and moDDC cells, as well as dampened ability of dendritic cells to activate differentiation of T cells. Consequently, it indicated that the lower retinoic acid concentration in lesion skin of K14-WFDC12 transgenic mice may activate DC cells and Th1 cell differentiation by affecting the expression of RAR-RXR transcription-related genes.

An increase in retinoic acid content can diminish immune cell activation during the onset and progression of psoriasis. Retinoic acid is the first-line therapy for the treatment of psoriasis according to current clinical medication guidelines, and the substance has a durable therapeutic effect on psoriasis. Our experimental results showed that the infiltration of DC cells and Th1 cells in the back skin lesions and lymph nodes of K14-WFDC12 transgenic mice was increased, as did the release of inflammatory cytokines IL-12 and IFN-γ, and the retinoic acid-related signaling pathway was altered. We speculated the downregulation of retinoic acid production in skin lesions on the psoriasis-like transgenic mice most likely increased immune cell differentiation and exacerbated inflammation. And the decreased concentration of retinoic acid may have resulted in the downregulation of RDH10 and DHRS9 and the upregulation of CRABP1 expression of the back skin lesions of K14-WFDC12 transgenic mice, thereby regulating the expression of IL-12 in dendritic cells and inducing Th1 cells of differentiation. The activation of immune cells was likely to cause changes in the expression of RAR-RXR transcriptional regulation genes by regulating the retinoic acid signaling pathway, boosting the maturation of LCs and moDDC cells, and initiating T-cell differentiation in our experimental model.

Protease inhibitors can affect the synthetic and metabolic signaling pathways of retinoic acid and regulate gene expression. Studies have shown that when myeloid leukemia cells were co-cultured with HIV-1 protease inhibitor and ATRA, HIV-1 protease inhibitor can up-regulate the expression of C/EBPϵ messenger RNA in the retinoic acid signaling pathway, thereby enhancing the inhibition role of ATRA on the induction immune cells differentiation, which was induced by myeloid leukemia cell (58). In the next step, we will use Th1 cytokine blockers or retinoic acid signal pathway modulators to observe whether the severity of psoriatic lesions in mice is improved after treatment, so as to further clarify the role of WFDC12 in the pathogenesis of psoriasis. Through proteomics, we predicted that CRABP1, DHRS9, and RDH10 genes might be most closely related to the function of WFDC12 or the retinoic acid pathway. We will further study the correlation in later studies. Further WFDC12 knockout mice are needed to verify whether the loss of WFDC12 has a protective effect on psoriatic lesions. *In vitro* experiments demonstrated the regulatory pathway or axis of WFDC12 using potential gene dysfunction in KCs. Although omics studies have shown that the retinoic acid pathway may be one of the pathways of action of WFDC12, we will further verify the expression of other pathways in KCs, such as immunity, protein folding, proliferation, and apoptosis.

In conclusion, WFDC12 might affect the activation of the retinoic acid signaling pathway and regulate the infiltration of DC cells in the skin lesions and lymph nodes, thereby inducing Th1 cells differentiation and increasing the secretion of IFN-γ to exacerbate psoriasis in mice. Our study provided a strong evidence for the regulation mechanism of WFDC12 in psoriasis developing, which would be conducive to treatment of psoriasis. However, future research needs to focus on the exploration of the specific mechanism by which WFDC12 protease inhibitor molecules regulate the retinoic acid synthesis and metabolism signaling pathways in the occurrence and development of psoriasis and confirm whether it relies on regulation genes expression by RAR-RXR transcriptional modulation.

## 4 Material and method

### 4.1 Animals

The K14-WFDC12 transgenic mice were commissioned to construct by Saiye Biotechnology Co., Ltd. The plasmid was shown in **Figure S1A**. C57BL/6 mice (wild type) used for breeding were purchased according to the policies and agreements approved by Sichuan University. Sterile house condition, 12 h light/12 h dark cycle, the temperature of 25 ± 1°C, and free access to water and food. The experiments were carried out following the National Institutes of Health’s ethical guidelines for the care and use of laboratory animals and the International Association for the Study of Pain (IASP). Collect the tail biopsies of mice and use the Mouse Direct PCR Kit (Bimake, B40015) for identification of genotype by polymerase chain reaction (PCR) **(** **Figure S1C** **)**. The mice with a 551-bp band in nucleic acid gel electrophoresis were identified as K14-WFDC12 transgenic mice, which specifically high-expressed WFDC12 protein in KCs (**Figure S1C**). The WFDC12 primer: F 5′-TCCAATTTACCCGAGCACCTTC-3′, R 5′-AGCCAGAAGTCAGATGCTCAAGG-3′. All experimental procedures were performed following the guidelines of experimental animals from Sichuan University.

### 4.2 Human subjects

Biopsies of lesional skin of four psoriasis patients and healthy skin of four donors were taken from the West China Hospital of Sichuan University. The screening samples were carried out in the same way as described before (18). This study was conducted by the principles of the Helsinki Declaration and was approved by the ethics committee of West China Hospital of Sichuan University (Chengdu, Sichuan, China).

### 4.3 GEO expression datasets

The datasets used in the analyses were obtained from the GEO database, which is an international public repository for high-throughput microarray and next-generation sequencing functional datasets. Three datasets were analyzed for exploring the expression of WFDC12 protein in lesion/non-lesional psoriasis patients and normal skin of healthy persons. In the GDS4602, a total of 180 skin tissue samples, containing 58 patients with psoriasis (lesion and non-lesion tissues from each psoriasis patient) and 64 healthy individuals, were contained for Affymetrix HU133 Plus 2.0 microarray analysis with more than 54,000 gene probes. Similarly, GDS4600 has 170 samples (85 samples for each class) and was created using the same methodology of Affymetrix Human Genome U133 Plus 2.0 Array. In the GDS5420, analysis results were obtained from lesional/non-lesional psoriatic skins for up to 43 days after treatment with different dosages of Brodalumab, which specially binds to and inhibits signaling *via* IL-17RA.

### 4.4 Imiquimod (IMQ)–induced psoriasis-like skin inflammation

The treatment of each group was as follows: The female mice (aged 8 weeks, 17–18 g) were sorted out to construct an IMQ-induced psoriasis model. Before induction of IMQ, the back skin of mice was shaved and exposed with an area of 2 cm × 3 cm. Following that, the backs of the mice were treated with Aldara cream (Sichuan MingXin Pharmaceutical Co., LTD., Sichuan, China) containing 5% IMQ once daily for 1–5 days. On the fifth day, the treated mice were photographed and sacrificed, then collect lesional area skins were for the following experimental analysis. Untreated mice were not used with any drugs and photographed, and the skin samples were collected directly on the fifth day.

### 4.5 PASI score

PASI refers to the area and severity of psoriasis and is a common scoring method for clinical psoriasis area and severity. Here, we refer to the scoring standards for psoriasis scores in K14-WFDC12 transgenic mice and WT mice, as follows: the degree of the scale, the degree of erythema, and the degree of thickening of the back skin; each factor is independent in the range of 0 to 4. Score (0—none, 1—slight, 2—moderate, 3—marked, 4—maximum, and recorded every 24 h). The total score ranges from 0 to 12.

### 4.6 Reverse transcription-quantitative polymerase chain reaction (RT-qPCR)

Mice skin tissues were collected, and TRIzol (Invitrogen; Thermo Fisher Scientific, Inc., Carlsbad, USA) was used to extract the total RNA according to the manufacture's protocol. The total RNA (2 µg) was reverse transcribed into cDNA using the PrimeScript RT reagent kit with gDNA Eraser (Takara Bio, Inc., Otsu, Japan) at 42°C for 50 min and 85°C for 5 min according to the instructions. cDNA (20 ng) was subjected to qPCR analysis with TB Green™ Premix Ex Taq™ II (Tli RNaseH Plus; Takara Bio, Inc., Otsu, Japan). PCR was run under the following conditions: initial denaturation at 95°C for 30 sec, 35 cycles of 95°C for 5 sec, annealing and extension at 60°C for 30 sec, and final extension at 72°C for 5 min. β-actin was used as the internal control, and quantification was performed using the 2−ΔΔCt method. All the RT-qPCR primers were purchased from Chengdu Qing Ke Zi Xi Biotechnology Co. and were listed in **Table S1**.

### 4.7 Western blot

The samples derived from skin tissue were lysed, separated by electrophoresis on SDS-PAGE gels (Beyotime Institute of Biotechnology, P0012AC) and transferred to polyvinylidene fluoride (PVDF) membranes (MerckMinipore, IPVH00010). For Western blotting detection, the proteins were incubated overnight with the following primary antibody: HA (Cell Signaling Technology, 1:1000 dilution), incubated overnight. Labeling of the primary antibodies was detected using goat anti-rabbit antibody conjugated to horseradish peroxidase (HRP) (Invitrogen,2215587,1:10000 dilution) and further detected using ECL reagents (MerckMinipore, WBULS0500). ImageJ was used for further quantification of the band intensities in the images, and only the band intensities in the linear range were included.

### 4.8 Hematoxylin and eosin (H&E) staining, microscopy, and image analysis

Human skin and mouse dorsal skin were fixed in 4% paraformaldehyde in PBS, embedded in paraffin, sectioned, and stained with H&E for histopathologic examination. Images were captured using an Olympus BX600 microscope (Olympus Corporation, Tokyo, Japan) and SPOT Flex camera (Olympus Corporation, Tokyo, Japan) and were analyzed with ImagePro Plus (version 6.0, Media Cybernetics) software. The epithelial thickness and infiltrating cells were evaluated in independent regions. For the measurement of skin thickness, 2–3 visual fields and 5–10 measuring points were selected for each back film, and the average value was taken.

### 4.9 Immunohistochemistry, microscopy, and image analysis

Human skin were fixed in 4% paraformaldehyde in PBS, and the fixed sections were incubated in 3% H_2_O_2_ solution in PBS at room temperature for 10 min. Antigen retrieval was performed in sodium citrate buffer (0.01 M, pH 6.0) in a microwave oven at 1000 W for 3 min. Nonspecific antibody binding was blocked by incubation with 5% normal goat serum in PBS for 1 h at room temperature. Slides were stained overnight at 4°C with the following primary antibodies: WFDC12 (proteintech, 25101-1-AP;1:500 dilution). The slides were subsequently washed and incubated with biotin-conjugated secondary antibodies for 30 min, and then with Horseradish Peroxidase Streptavidin (HRP Streptavidin) for 30 min (SPlink Detection Kits; ZSGB-BIO, SP-9001 or SP-9002). The sections were developed using the 3,3ʹ-Diaminobenzidine (DAB) substrate kit (ZSGB-BIO, ZLI- 9017) and counterstained with hematoxylin. Images were captured using an Olympus BX600 microscope and SPOT Flex camera. ImagePro Plus was used for further quantification of the DAB intensity.

### 4.10 Histology, immunostaining, microscopy, and image analysis

Tissue biopsies were directly embedded in OCT compound, and the frozen sections were fixed in cold methanol-acetone (1:1) for 15 min, permeabilized with 0.3% Triton X-100 (Sigma-Aldrich, X100) in PBS for 15 min, blocked with 5% BSA (Sigma-Aldrich, B2064) in PBS for 30 min, and then incubated overnight at 4°C with primary antibodies against WFDC12 (proteintech, 25101-1-AP;1:500 dilution). As a secondary reagent, Goat Anti-Rabbit IgG H&L (Cy3 ^®^) preadsorbed (1:200 dilution, ab6939) were used; they were both from Invitrogen. Nuclear counterstaining was per- formed with 4ʹ,6-diamidino-2-phenylindole (DAPI; Sigma- Aldrich, D9542). Images were analyzed with a Leica DM RXA2 confocal microscope controlled by Leica Microsystems confocal software (version 2.61 Build 1537; all from Leica Microsystems, Wetzlar, Germany). ImageJ (National Institutes of Health) was used for further quantification of the fluorescence and intensities of the images.

### 4.11 Enzyme-linked immunosorbent assay (ELISA)

To detect the levels of IL17A in the serum of mouse, the serum were collected, and the IL17A levels were measured using the Mouse IL17A ELISA kit [NEOBIOSCIENCE, EMC008(H)] according to the manufacturer’s instructions.

### 4.12 Flow cytometry

#### 4.12.1 Single-cell suspension of epidermis

To obtain single-cell suspension from dorsal skin, samples were removed from the subcutaneous fat and mucosal tissue with the scalpel and spread into a petri dish.The samples were then incubated in 2.4 U/ml dispase II overnight at 4°C and then immersed in Dulbecco's Modified Eagle Medium (DMEM) containing 50% (v:v) FBS to inactivate the dispase II.Gently scrape off the epidermal layer and add 5 ml of 0.25% EDTA-free trypsin (Thermo Fisher, 15050057) to the tube to obtain a single cell suspension, after digestion at 37°C for 20 min. Finally, neutralized with 5 ml DMEM medium (GbicoTM, LS11995065). Single-cell suspension of epidermis cells was made followed by mechanical dissociation with a gentle MACS dissociator (Miltenyi Biotech, Bergisch Gladbach, Germany), and filtered sequentially through 70μm cell strainers (BD Bioscience, 352350), and cells were washed once with PBS.

#### 4.12.2 Single-cell suspension of lymph nodes

To obtain single-cell suspension from lymph nodes, samples were ground in 40-μm cell strainers (BD Bioscience, 352340) with 5 ml of PBS solution, and then filtered with 70-μm cell strainers (BD Bioscience, 352350). Cells were washed once with PBS.

#### 4.12.3 Analysis of LCs

The cells were washed and resuspended in PBS. The single-cell suspensions were stained with the following antibodies: CD207-PE (144204, BioLegend, San Diego, CA, USA), FITC-MHCII (I-A-I-E, 107606, BioLegend, San Diego, CA, USA), APC/Cy7-CD11c (117324, BioLegend, San Diego, CA, USA), PE/Cy7-CD86 (25-0862-82, eBioscience, Carlsbad, USA), APC-CD40 (17-0401-82, eBioscience, Carlsbad, USA), PerCP/Cy5.5-7-aminoactinomycin D (7-AAD) (BioLegend, 420404; 0.5 μg/ml, San Diego, CA, USA). Flow cytometry was performed using the NovoCyte flow cytometer and ACEA NovoExpress™ software (ACEA Biosciences, San Diego, CA, USA).

#### 4.12.4 Analysis of moDDCs

The cells were washed and resuspended in PBS. The single-cell suspensions were stained with the following antibodies: PerCP/Cy5.5-7-aminoactinomycin D (7-AAD) (BioLegend, 420404; 0.5 μg/ml, San Diego, CA, USA), APC-CD11b (17-0112-82, eBioscience, Carlsbad, USA), APC/Cy7-Ly6C (1208026, BioLegend, San Diego, CA, USA), BV510-CD45 (1033137, BioLegend, San Diego, CA, USA), BV711-CD64 (139311, BioLegend, San Diego, CA, USA), FITC-MHCII, PE-MerTK (151506, BioLegend, San Diego, CA, USA).

The BD LSRFortessaTM and Flow Jo™ software (BD Biosciences, Franklin. Lakes, NJ, USA) were used for flow cytometry analysis.

#### 4.12.5 Analysis of differentiation of Th1 and Th17 cells

For analyzing the expression of IFN-γ and IL-17, the single-cell suspensions were incubated for 3 h at 37°C with PMA (Sigma-Aldrich, p1585; 200 ng/ml, Missouri, USA), brefeldin A (BioLegend, 420601; 5 µg/ml, San Diego, CA, USA), and ionomycin (Abcam, ab120116; 1 µg/ml, Cambridge, UK). And then washed and stained with fixable viability stain 620 (FVS 620; BD-Biosciences, Franklin. Lakes, NJ, USA, 564996) for 10 min. Next, stain cells with following surface antibodies: APC/Cy7-CD3 (100222, BioLegend, San Diego, CA, USA), PerCP/Cy5.5-CD4 (100434, BioLegend, San Diego, CA, USA), PE/Cy7-CD8 (100722, BioLegend, San Diego, CA, USA). After performing surface staining as described above, 4% paraformaldehyde was used to fix cells and added PBS solution (containing 0.1% Triton X-100) to permeabilize the cell surface. Intracellular staining antibodies were included: PE-IL-17A (506903, BioLegend, San Diego, CA, USA), FITC-IFN-γ (505806, BioLegend, San Diego, CA, USA). After staining for 30 min, the cells were washed by PBS and using the NovoCyte flow cytometer and ACEA NovoExpress™ software (ACEA Biosciences, San Diego, CA, USA) for analysis.

#### 4.12.6 Analysis of IL-17A-producing γδT cells

The single-cell suspensions were incubated for 3 h at 37°C with PMA (Sigma-Aldrich, p1585; 200 ng/ml), brefeldin A (BioLegend, 420601; 5 µg/ml), and ionomycin (Abcam, ab120116; 1 µg/ml). And then washed and stained with fixable viability stain 620 (FVS 620; BD Biosciences, 564996) for 10 min. Next, stain cells with following surface antibodies: γδT-PE-CY5 (15-5711-81, eBioscience). After performing surface staining as described above, 4% paraformaldehyde was used to fix cells and added PBS solution (containing 0.1% Triton X-100) to permeabilize the cell surface. Intracellular staining antibodies were included: IL17A-APC (506916, BioLegend). After staining for 30 min, the cells were washed by PBS and using the NovoCyte flow cytometer and ACEA NovoExpress™ software (ACEA Biosciences, San Diego, CA, USA) for analysis.

### 4.13 Extract protein and quantification of protein lysis solution

The appropriate amount of each sample was weighed and transferred into a 2-ml centrifuge tube, add two steel beads, add 1X Cocktail with an appropriate amount of SDS, put on ice for 5 min, add DTT with the final concentration of 10 mM. Then, use a grinder (power is 60 HZ, time is 2 min) to crush the tissue, centrifuge at 25,000*g*, 4°C for 15 min, and collect the supernatant. Water bath at 56°C for 1 h after adding DTT with the final concentration of 10 mM again. The IAM at a final concentration of 55 mM was added and placed in a dark room for 45 min. Add cold acetone to the protein solution at a ratio of 1:5, place in a refrigerator at -20°C for 30 min, centrifuge at 25,000*g*, 4°C for 15 min, and discard the supernatant. Use a grinder (60 HZ, 2min) to promote air-dried protein solubilization in lysis buffer without SDS. Finally, centrifuge for 15 min at 25,000*g*, 4°C to take the supernatant, and the supernatant is the protein solution. The following quality control of protein extraction was performed by the Bradford method.

### 4.14 Isobaric tags for relative and absolute quantitation (iTRAQ)–based proteomics profiling

#### 4.14.1 iTRAQ labeling and peptide fractionation

Total proteins (100 μg) were removed from each mice sample solution, and the proteins were digested using trypsin-gold (Promega, Madison, WI, USA) at a protein (μg): trypsin(μg) ratio of 20:1, vortexed, centrifuged at low speed for 1 min, and then incubated for 2 h at 37°C. After trypsin digestion, the peptides were freeze-dried, re-dissolved in 0.5 M tetraethylammonium bromide (TEAB) for different iTRAQ reagents labeling (AB SCIEX, Framingham, MA, USA). Briefly, one unit of iTRAQ reagent was thawed and then reconstituted in 50 μl of isopropanol. And stand at room temperature for 2 h. Each sample (three samples of one group) of K14-WFDC12 transgenic mice and WT mice were labeled with iTRAQ reagents with molecular masses of 113, 114, 115, 116, 117, and 118 Da, respectively. After labeling, the pooled mixtures of the iTRAQ-labeled peptides were fractionated by strong cationic exchange (SCX) chromatography using an LC–20AB HPLC Pump system (Shimadzu, Kyoto, Japan). The iTRAQ-labeled peptide mixtures were subsequently reconstituted in the mobile phase (5% ACN, pH 9.8) and loaded onto a 4.6 mm × 250 mm Gemini C18 column for liquid phase separation of the sample. The peptides were eluted at a flow rate of 1 ml/min using the following gradient program: 5% mobile phase B (95% ACN, pH 9.8) for 10 min. Five percent to thirty-five percent mobile phase B for 40 min, 35–95% mobile phase B for 1 min, mobile phase B for 3 min, and 5% mobile phase B for 10 min. The elution peak was monitored at a wavelength of 214 nm and one component was collected per minute, and the samples were combined according to the chromatographic elution peak map to obtain 20 components, which were then freeze-dried.

#### 4.14.2 HPLC and Mass Spectrometry detection

The dried peptide samples were reconstituted with mobile phase A (2% ACN, 0.1% FA), centrifuged at 20,000*g* for 10 min, and the supernatant was taken for injection. Separation was performed by Thermo UltiMate 3000 UHPLC. The sample was first enriched in trap column and desalted, and then entered a self-packed C18 column (75-um inner diameter, 3 µm column > particle size, 25 cm column length) and separated at a flow rate of 300 nl/min by the following effective gradient: 0–5 min, 5% mobile phase B (98% ACN, 0.1% FA); 5–45min, mobile phase B linearly increased from 5% to 25%; 45–50 min, mobile phase B increased from 25% to 35%; 50–52 min, mobile phase B rose from 35% to 80%; 52–54 min, 80% mobile phase B; 54–60 min, 5% mobile phase B. The nanoliter liquid phase separation end was directly connected to the mass spectrometer.

The peptides separated by liquid-phase chromatography were ionized by a nanoESI source and then passed to a tandem mass spectrometer Q-Exactive HF (Thermo Fisher Scientific, San Jose, CA) for DDA (data-dependent acquisition) mode detection. The main parameters were set: ion source voltage was set to 1.9 kV; MS1 scanning range was 350~1600 m/z; the resolution was set to 60,000; MS2 starting m/z was fixed at 100, the resolution was 15,000. The ion screening conditions for MS2 fragmentation: charge 2^+^ to 6^+^, and the top 30 parent ions with the peak intensity exceeding 10,000. The ion fragmentation mode was HCD, and the fragment ions were detected in Orbitrap. The dynamic exclusion time was set to 30 s. The AGC was set to MS1 3E6, MS2 1E5.

#### 4.14.3 PPI networks construction

STRING v10.1 (http://string-db.org/) was applied to analyze the PPI of DEPs identified in the current study and to construct PPI networks. The protein interaction information was extracted from the differential genes of lesion areas of K14-WFDC12 transgenic mice and WT mice within the IMQ-induced psoriasis model. The active prediction methods, such as database, experiment, and text mining, were enabled, as well as species limited to “Mus musculus” and an interaction score > 0.7 were applied to construct the PPI networks.

#### 4.14.4 KEGG pathway analysis

The Database for Annotation, Aisualization and Integrated Discovery (DAVID) v6.8 comprises a full knowledgebase update to the KEGG pathways of web-accessible programs (https://david.ncifcrf.gov). We utilize the KEGG pathway analysis of lesion areas of K14-WFDC12 transgenic mice and WT mice within IMQ-induced psoriasis model to acquire pathway information of DEGs.

### 4.15 Statistics

The statistical software GraphPad Prism 9.0 was used for data analysis in the experiment. The difference between the two groups was compared by unpaired or paired *t*-test, and the experimental data were expressed as *M* ± *SD*. *p < 0.05, the difference is statistically significant, **p < 0.01, the difference is statistically very significant, ***p < 0.001, the difference is statistically extremely significant, and ns indicates no statistical significance.

## Data availability statement

The mass spectrometry proteomics data have been deposited to the ProteomeXchange Consortium (http://proteomecentral.proteomexchange.org) *via* the iProX partner repository with the dataset identifier PXD036247.

## Ethics statement

The present study was performed in accordance with the principles of the Helsinki Declaration and approved by the Ethics Committee of the West China Hospital, Sichuan University (Chengdu, Sichuan, China). Written informed consent was obtained from all the study participants prior to the study.The animal study was reviewed and approved by Institutional Animal Care and Treatment Committee of Sichuan University (Chengdu, PR China).

## Author contributions

FZh, CZ, and JL designed the experiments and present study. FZh and CZ performed most of the experiments and wrote the draft of manuscript. GL, HPZ, LG, HZ, YX, ZW, JY, YwH, FZe, XW, QZ, JH, CY, PZ, NH, YH, WW, KC, and WL contributed to data analyses and rechecked the manuscript and put forward meaningful comments on it. All authors approved the final version of the manuscript.

## Funding

This work was supported by the National Natural Science Foundation of China (81472650, 81673061, 31271483, 81573050, 31872739, 30300313,82003358); National Science and Technology Major Project (2019ZX09201003-003, 2018ZX09733001-001-006, 2013ZX09301304001-003, 2012ZX10002006–003–001, 2009ZX09103-714); Sichuan Provincial Outstanding Youth Fund (2015JQO025); Key Research and Development Program of Sichuan Province (2020YFS0271); Applied Basic Research Program of Sichuan Province (2008SZ0093).

## Acknowledgments

The authors thank WL from the West China Hospital for providing support and discussions.

## Conflict of interest

The authors declare that the research was conducted in the absence of any commercial or financial relationships that could be construed as a potential conflict of interest.

## Publisher’s note

All claims expressed in this article are solely those of the authors and do not necessarily represent those of their affiliated organizations, or those of the publisher, the editors and the reviewers. Any product that may be evaluated in this article, or claim that may be made by its manufacturer, is not guaranteed or endorsed by the publisher.

## Abbreviation

ATRA, all-trans retinoic acid; TF AP-1, transcription factor AP-1; CRABP1, cellular retinoic acid binding protein 1; cDCs, conventional dendritic cells; CLDN1, Claudin 1; CLDN4, Claudin 4; DHRS9, ehydrogenase/Reductase 9; Flg, Filaggrin; GEO, Dataset Gene Expression Omnibus Dataset; IOD, integrated option density; IMQ, Imiquimod; iTRAQ, isobaric tags for relative and absolute quantitation; iNOS, iducible nitric oxide synthase; KCs, keratinocytes; LCP2, lymphocyte cytosolic protein 2; LGALS1, lectin, galactoside-binding, soluble, 1; MHC II, major histocompatibility complex II; MDCs, myeloid dendritic cells; moDDCs, monocyte-derived dendritic cells; pDCs, plasmacytoid dendritic cells; PPI, protein–protein interaction; Th1 cells, T helper type 1 cells; Th17 cells, T helper type 17 cells; RA, retinoic acid; RDH10, retinol dehydrogenase 10; SLPI, secretory leukocyte peptidase inhibitor; Th cells, helper T cells; TLR7, Toll-like receptor 7; VIM, Vimentin; WFDC protein, WAP four-disulfide core domain protein; WFDC12 protein, WAP four-disulfide core domain protein 12.
