# Supplementary material for: A role for whey acidic protein four-disulfide-core 12 (WFDC12) in the pathogenesis and development of psoriasis disease
Source: Front Immunol. 2022 Sep 6;13:873720. doi: 10.3389/fimmu.2022.873720 (PMC9485559; doi:10.3389/fimmu.2022.873720)
Supplement: Supplementary file 1 [file DataSheet_1.doc]

**A role for whey acidic protein four-disulfide-core 12(WFDC12) in the pathogenesis and development of psoriasis disease**

Fulei Zhao^1*^,Chen Zhang^1*^,Guolin Li^1*^,Huaping Zheng^1^,Linna Gu^1^,Hong Zhou^1^,Yuanyuan Xiao^2,3^,Zhen Wang^1^,Jiadong Yu^1^,Yawen Hu^1^, Fanlian Zeng^1^,Xiaoyan Wang^1^, Qixiang Zhao^1^, Jing Hu^1^, Chengcheng Yue ^1^, Pei Zhou^1^, Nongyu Huang^1^ ,Yan Hao^1^, ﻿Wenling Wu^1^,Kaijun Cui^4^,Wei Li^5^, Jiong Li^1#^.

1 State Key Laboratory of Biotherapy and Cancer Center, West China Hospital, West China Medical School, Sichuan University and Collaborative Innovation Center for Biotherapy, Chengdu, China.

2 Department of Obstetrics and Gynecology, West China Second Hospital of Sichuan University, Chengdu, P.R. China.

3 Key Laboratory of Birth Defects and Related Diseases of Women and Children, Sichuan University, Ministry of Education, Chengdu, P.R. China

4 Department of Cardiology, West China Hospital, Sichuan University, Chengdu, China

5 Department of Dermatology, Rare Diseases Center , West China Hospital, Sichuan University, Chengdu, China.

^*^These authors contributed equally to this work.

^#^corresponding author.

Jiong Li, State Key Laboratory of Biotherapy and Cancer Center, West China Hospital, West China Medical School, Sichuan University and Collaborative Innovation Center for Biotherapy, Chengdu, China.

Email: lijionghh@scu.edu.cn

**Abstract**

WAP four-disulfide core domain protein 12(WFDC12) has been implicated in the pathogenesis of psoriasis but the specific molecular mechanism is not clearly defined. In this study, we found the expression of WFDC12 protein closely correlated with psoriasis. WFDC12 in Keratinocyte might increase infiltration of Langerhans (LCs) and monocyte-derived dendritic cells (moDDCs), upregulating the co-stimulation molecular CD40/CD86. Th1 cells in lymph nodes were higher in K14-WFDC12 transgenic psoriasis-like mice. Meanwhile, the mRNA of IL-12 and IFN-γ in the lesion skin was significantly increased in transgenic mice. Moreover, we found that the expression of the proteins that participated in the retinoic acid-related pathway and immune signaling pathway was more changed in the lesion skin of K14-WFDC12 transgenic psoriasis-like mice. Collectively, the results implied that WFDC12 might affect the activation of the retinoic acid signaling pathway and regulate the infiltration of DC cells in the skin lesions and lymph nodes, thereby inducing Th1 cells differentiation and increasing the secretion of IFN-γ to exacerbate psoriasis in mice.

**Keywords**: psoriasis, keratinocytes, WFDC12, inflammation, retinoic acid

**
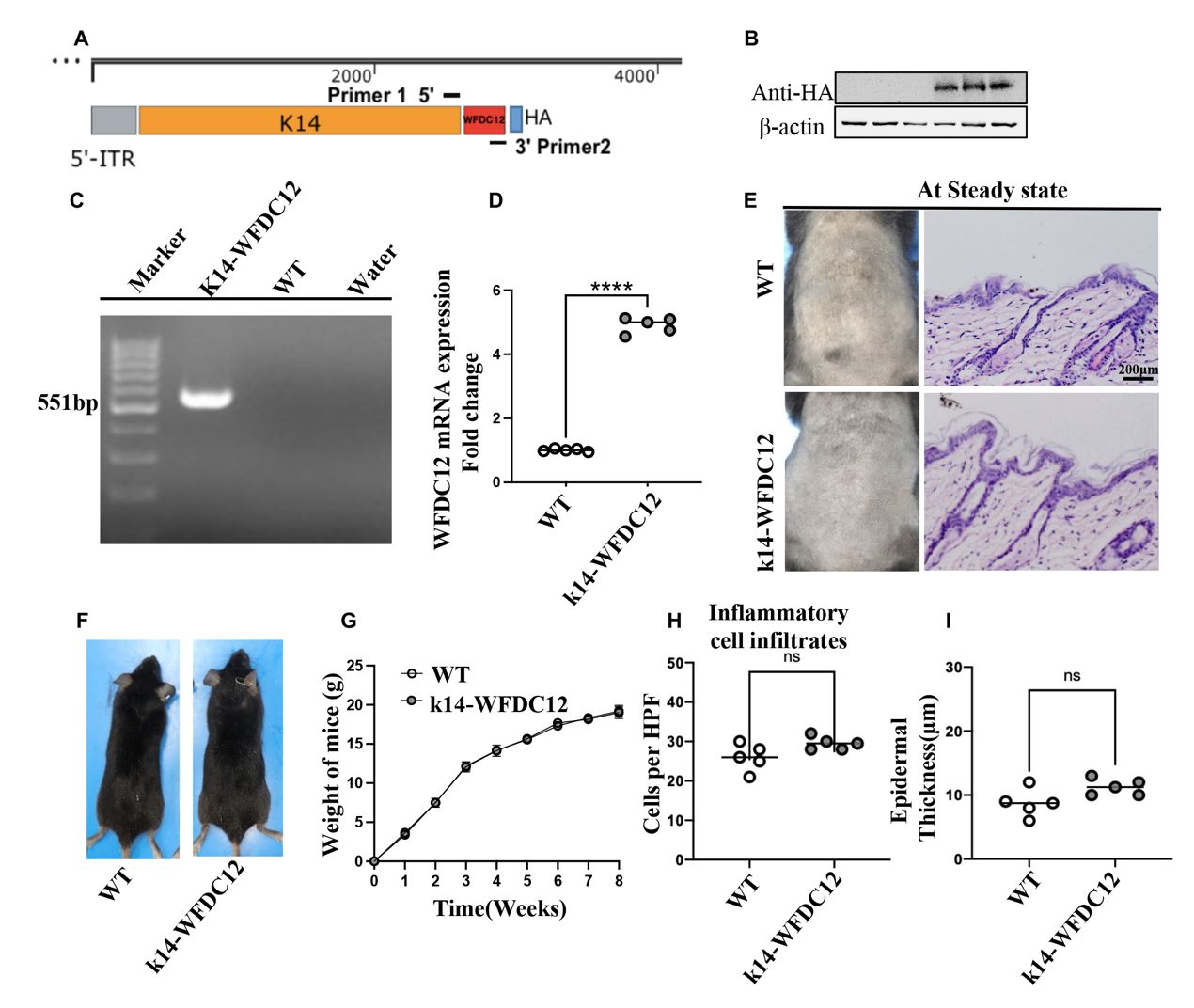
**

**Figure. S1** **Transgenic mice construction and evaluation** **in K14-WFDC12 transgenic mice and WT mice at steady state.** **(A)** The plasmid construction map of skin-specific expression of WFDC12 transgenic mice. **(B)** Western blot to confirm that WFDC12 protein is successfully over-produced in epidermal keratinocytes in transgenic mice, n=3/group. **(C)** Genotype identification of K14-WFDC12 transgenic mice. **(D)** Quantification of WFDC12 mRNA expression in back skin of K14-WFDC12 transgenic mice and WT mice, n=4/group. **(E)** H&E staining of paraffin sections of skin tissue at steady state, n=5/group. **(F)** Appearance of transgenic mice and WT mice at steady state. **(G)** statistics of weight of mice at steady state, n=5/group. **(H)**The number of inflammatory cell infiltrates in the dermis at steady state. Numbers of dermal inflammatory cells were counted per high-power field from five mice per group. **(I)** epidermal thickness statistics of mice at steady-state, n=5/group. **(G)** Unpaired t-test was applied to assess the significance of difference between the two groups at the same time points. **(D,H,I)** unpaired Student’s t-test.All the data shown in this and the figures below came from samples from individual mice unless stated otherwise. The data are presented as Mean ± SD. **** p<0.0001. ns, not significance.

**
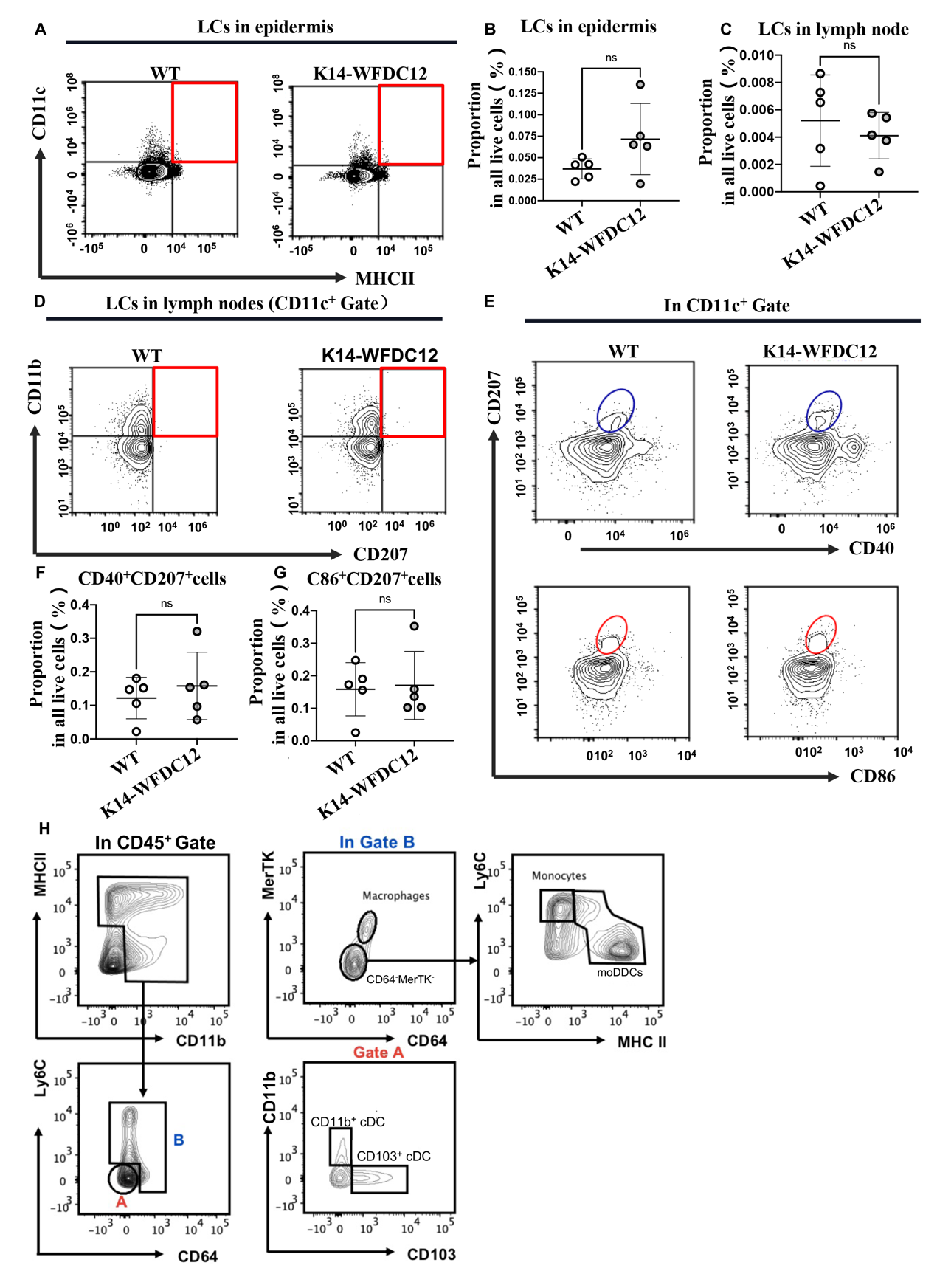
**

**Figure. S2** **Infiltration of LCs and moDDCs be detected by Flow cytometry in K14-WFDC12 transgenic mice and WT mice at steady state.** **(A, B)** Representative and quantiﬁcation of FACS analysis of LCs in the epidermis, n=5/group. **(C, D)** Representative and quantiﬁcation of FACS analysis of LCs in lymph nodes, n=5/group.**(E, F, G)** Representative and quantiﬁcation of FACS analysis of CD40/CD86 expression of LCs in the epidermis, n=5/group. **(****H)** strategy of Flow cytometry to identify monocytes and moDDCs in lymph node. All the data shown in this and the figures below came from samples from individual mice unless stated otherwise. The data are presented as Mean ± SD. ns, not significance (unpaired Student’s t-test).


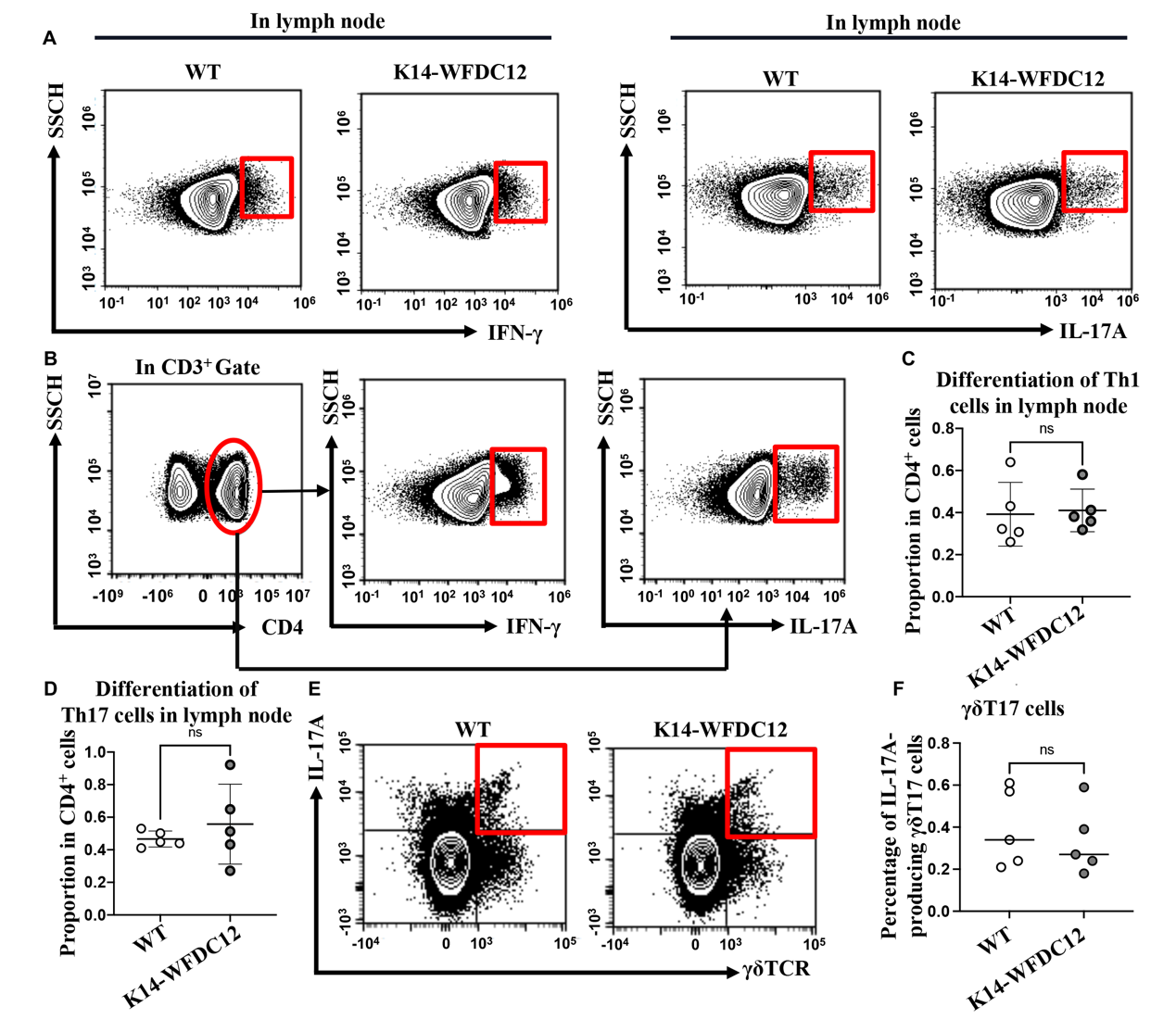


**Figure. S3 Th1 、Th17 and IL-17A-producing γδT17 cells be detected in K14-WFDC12 transgenic mice and WT mice at steady state. (A)** Representative of Th1 cells secreting IFN-γ and IL-17-producing cells (Th17 cells) in lymph nodes of mice. **(B)** Strategy of Flow cytometry to identify Th1 and Th17 cells in lymph node. **(C, D)** Quantification of Th1 cells and Th17 cells in lymph nodes of mice (proportion of CD4^+^ cells Gate) ,n=5/group. **(E, F)** Representative FCM images and quantification indicated percentages of IL-17A-producing γδT17 cells in lymph nodes of K14-WFDC12 transgenic mice and WT mice, n=5/group. All the data shown in this and the figures below came from samples from individual mice unless stated otherwise. The data are presented as Mean ± SD. ns, not significance (unpaired Student’s t-test).


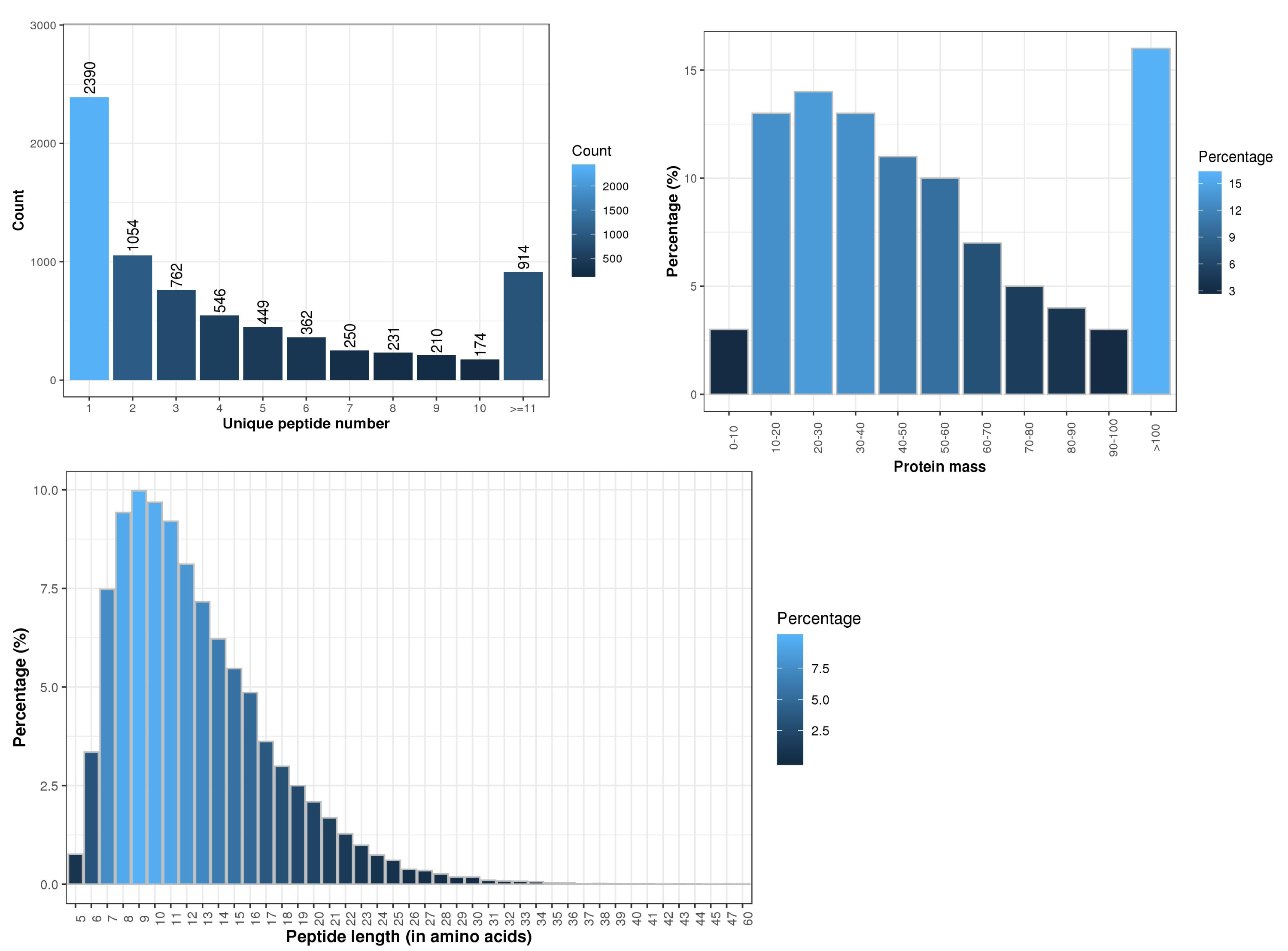


**Figure. S4 The Peptide length, number and protein mass of the identified proteins in different mice samples.**

**Table.S1** **Primer sequences used for** **RT-qPCR**

| Gene(species) | forward | reverse |
| --- | --- | --- |
| DHRS9(mouse) | AAAGCTCGAGGGCGTGTTAT | AGTATAGCCCCCTCCACCAA |
| CRABP1(mouse) | GAGGGGGATGGCCCTAAAAC | CATCATCGGCGCCAAATGTC |
| RDH10(mouse) | AAATCCTGCCCCCGTGTAAC | CTCCTTGCGGACCCTTTCAG |
| WFDC12(human) | TGTGTCAAGGCCATACCCTG | AGGTGCCAAGTGAGAGTGAC |
| WFDC12(mouse) | GGAGAAAAGAGTTTGCCCGC | GCACGCATTTGAAGCCACAT |
| β-actin（human） | CCACGAAACTACCTTCAACTCC | GTGATCTCCTTCTGCATCCTGT |
| β-actin(mouse) | CCTCTATGCCAACACAGTGC | ACATCTGCTGGAAGGTGGAC |
| IFN-γ(mouse) | TCAAGTGGCATAGATGTGGAAGAA | TGGCTCTGCAGGATTTTCATG |
| CCL19(mouse) | CATAAATTGGAGCTGGTGGCAG | AGGAGCCAAGTGCAAGTGAG |
| IL-17A(mouse) | CTCAGACTACCTCAACCGTTCC | CATGTGGTGGTCCAGCTTT |
